# Boceprevir or Telaprevir Based Triple Therapy against Chronic Hepatitis C in HIV Coinfection: Real-Life Safety and Efficacy

**DOI:** 10.1371/journal.pone.0125080

**Published:** 2015-04-29

**Authors:** Karin Neukam, Daniela I. Munteanu, Antonio Rivero-Juárez, Thomas Lutz, Jan Fehr, Mattias Mandorfer, Sanjay Bhagani, Luis F. López-Cortés, Annette Haberl, Marcel Stoeckle, Manuel Márquez, Stefan Scholten, Ignacio de los Santos-Gil, Stefan Mauss, Antonio Rivero, Antonio Collado, Marcial Delgado, Juergen K. Rockstroh, Juan A. Pineda

**Affiliations:** 1 Unit of Infectious Diseases and Microbiology, Valme University Hospital and Seville Institute of Biomedicine (IBiS), Seville, Spain; 2 RIS-HEP07 Study Group of the Spanish AIDS Research Network; 3 Department of Medicine I, Bonn University Hospital, Bonn-Venusberg, Germany; 4 Matei Bals National Institute of Infectious Diseases, Bucharest, Romania; 5 Unit of Infectious Diseases, Reina Sofía University Hospital, Maimónides Institute of Biomedical Investigation of Cordoba (IMIBIC), Cordoba, Spain; 6 Infektiologikum Frankfurt, Frankfurt/Main, Germany; 7 Division of Infectious Diseases & Hospital Epidemiology, University Hospital Zurich, University of Zurich, Zurich, Switzerland; 8 Division of Gastroenterology and Hepatology, Department of Internal Medicine III, Medical University of Vienna and Vienna HIV & Liver Study Group, Vienna, Austria; 9 Department of Infectious Diseases/HIV Medicine, Royal Free London NHS Foundation Trust, London, United Kingdom; 10 Infectious Diseases, Microbiology and Preventive Medicine, Virgen del Rocío University Hospital and Seville Institute of Biomedicine (IBiS), Seville, Spain; 11 Department of Medicine II, Frankfurt University Hospital, Frankfurt/Main, Germany; 12 Division of Infectious Diseases & Hospital Epidemiology, University Hospital Basel, Basel, Switzerland; 13 Unit of Infectious Diseases, Virgen de la Victoria University Hospital, Malaga, Spain; 14 Praxis Hohenstaufenring, Cologne, Germany; 15 Infectious Diseases Unit, La Princesa University Hospital, Madrid, Spain; 16 Center for HIV and Hepatogastroenterology, Dusseldorf, Germany; 17 Infectious Diseases Unit, Torrecardenas University Hospital, Almeria, Spain; 18 Unit of Infectious Diseases, Carlos Haya Regional University Hospital, Malaga, Spain; Harvard Medical School, UNITED STATES

## Abstract

**Background and Aims:**

Clinical trials of therapy against chronic hepatitis C virus (HCV) infection including boceprevir (BOC) or telaprevir (TVR) plus pegylated interferon and ribavirin (PR) have reported considerably higher response rates than those achieved with PR alone. This study sought to evaluate the efficacy and safety of triple therapy including BOC or TVR in combination with PR in HIV/HCV-coinfected patients under real-life conditions.

**Methods:**

In a multicentre study conducted in 24 sites throughout five European countries, all HIV/HCV-coinfected patients who initiated a combination of BOC or TVR plus PR and who had at least 60 weeks of follow-up, were analyzed. Sustained virologic response 12 weeks after the scheduled end of therapy date (SVR12) and the rate of discontinuations due to adverse events (AE) were evaluated.

**Results:**

Of the 159 subjects included, 127 (79.9%) were male, 45 (34.4%) were treatment-naïve for PR and 60 (45.4%) showed cirrhosis. SVR12 was observed in 31/46 (67.4%) patients treated with BOC and 69/113 (61.1%) patients treated with TVR. Overall discontinuations due to AE rates were 8.7% for BOC and 8% for TVR. Grade 3 or 4 hematological abnormalities were frequently observed; anemia 7%, thrombocytopenia 17.2% and neutropenia 16.4%.

**Conclusion:**

The efficacy and safety of triple therapy including BOC or TVR plus PR under real-life conditions of use in the HIV/HCV-coinfected population was similar to what is observed in clinical trials. Hematological side effects are frequent but manageable.

## Introduction

Treatment strategies aimed to enhance rates of sustained virologic response (SVR) are of highest priority in patients with chronic hepatitis C and HIV coinfection, since SVR dramatically reduces the incidence of hepatic decompensations and mortality in this setting [1,2]. Until 2009, standard-of-care for treating chronic hepatitis C virus (HCV) infection consisted of dual therapy with pegylated interferon (peg-IFN) plus ribavirin (RBV). However, in the setting of HIV/HCV genotype 1-coinfected patients, SVR rates with dual therapy did not exceed 25% in clinical practice [3]. The arrival of direct acting antivirals (DAA) against HCV has ushered in a new era in the management of chronic hepatitis C. Considerable increases of SVR to triple combination of the first generation HCV-protease inhibitors (PI) boceprevir (BOC) or telaprevir (TVR) with peg-IFN and RBV in the HCV-monoinfected population have been reported in pivotal clinical trials in HCV-monoinfected and in HIV/HCV-coinfected subjects [4–11]. As a consequence, in 2012, triple therapy including BOC or TVR in addition to peg-IFN and RBV became the standard therapy for patients coinfected with HIV and HCV genotype 1 in Europe [12]. Although current recommendations include next-generation DAA [13], first generation PIs are still widely used in some countries due to financial constraints.

However, the use of first-generation HCV PIs raises important concerns, such as tolerability, increased cost, drug-drug interactions, and decreased adherence due to a high pill burden and multiple dosing. Furthermore, data on safety and efficacy of DAA-based therapies are derived from clinical trials and the so-called “real-life” studies are actually based on early access programmes or compassionate use programmes [8,9,14–17]. The sparse data derived from studies conducted in real-life settings mainly come from HCV-monoinfected patients [18–22]. Some of these studies report evidence of reduced tolerability and efficacy of BOC and TVR-based treatments as observed in pivotal trials. However, the population characteristics of these studies are diverse and response rates and safety profiles of DAAs may not be comparable between HCV-monoinfected and HIV/HCV-coinfected patients. In this context, the coinfected population has specific issues such as the co-administration of antiretroviral therapy (ART), which increases the pill-burden even further and may cause drug-drug interactions, making modification of ART or TVR dose adjustment necessary in a high number of patients. Still, to date there is no study available to evaluate this issue in large populations of HIV/HCV-coinfected patients and under real-life conditions. Data on the efficacy and tolerability of first-generation DAA-based regimens in the clinical practice are needed.

Therefore, the aim of this study was to determine the efficacy and safety of triple therapy including BOC or TVR plus peg-IFN and RBV in patients with chronic hepatitis C and HIV coinfection in routine clinical practice.

## Patients and Methods

### Patients and study design

All patients from populations prospectively followed in 24 health care settings from Austria, Germany, Spain, Switzerland and the United Kingdom were included in this observational study if they were i) older than 18 years, ii) coinfected with HIV, iii) were given triple therapy against HCV including BOC or TVR in combination with Peg-IFN plus RBV, according to the standard of care for chronic hepatitis caused by HCV genotype 1 at the time of starting therapy and iv) started therapy before January 2013 in order to have a minimum follow-up of 60 weeks, so that SVR 12 weeks after ending therapy might be evaluated. Key exclusion criteria were participation in a clinical trial or having received prior DAA-based therapy. Visits were scheduled at least at treatment weeks (TW) zero, four, 12, 24, and 48, as well as 12 weeks after the scheduled end of treatment (EOT). Those patients who had a four-week lead-in phase with Peg-IFN/RBV alone were additionally seen at TW8. At each visit, plasma HCV-RNA was quantified and hematological parameters were determined. Management of adverse events (AE), as well as the decision to discontinue therapy due to side effects, was carried out according to the criteria of caring physicians.

### Treatment duration, dosing and definition of response

The scheduled treatment duration for all patients was 48 weeks. Patients received a three-drug combination based on Peg-IFN alfa-2a or Peg-IFN alfa-2b at a dose of 180 μg or 1.5 μg/kg once per week, respectively, and oral RBV at daily doses of 800–1200 mg. Treatment duration and futility rules were in accordance with the package insert of BOC and TVR and international guidelines [23–25]. BOC was administered orally at doses of 800 mg every eight hours for 44 weeks. Oral TVR was given at doses of 750 mg every eight hours or 1125 mg twice a day during the first 12 weeks of therapy, or from TW5 to TW16 in patients who underwent a lead-in phase first with Peg-IFN/RBV alone, followed by Peg-IFN/RBV dual therapy until reaching the scheduled EOT. Non-response to the respective DAA was assumed when the stopping rules were met: all treatment was discontinued and non-response to BOC was assumed when plasma HCV RNA was >100 IU/mL at TW12 or when HCV RNA was detectable at W24. TVR-based therapy was discontinued and non-response was assumed if plasma HCV RNA was >1000 IU/mL four or 12 weeks after TVR initiation, or if it was detectable at TW24For both HCV PIs, an increase of HCV RNA ≥1 log_10_ IU/mL following a decline, as well as detectable HCV RNA following undetectability, were considered viral breakthrough (VB) and, consequently, treatment was stopped. Detectable HCV RNA at the SVR12 evaluation time point after achieving EOT response was considered relapse. SVR12 was defined as undetectable HCV-RNA 12 weeks after scheduled EOT.

### Definition of previous response and hepatic fibrosis

Null response to previous interferon-based therapy was considered when patients had not achieved early virological response (EVR; a 2 log_10_ decline of HCV RNA or HCV RNA undetectable) at treatment week 12, while partial response was defined as having achieved EVR at week 12 without reaching undetectable HCV RNA in week 24. Patients who had achieved undetectable HCV RNA but had detectable HCV RNA at the EOT were defined as having suffered VB while those who had undetectable HCV RNA at the EOT but subsequently showed detectable HCV RNA at week 24 post-treatment were considered previous relapsers. Cirrhosis was determined by liver biopsy and defined as F4 according to the Scheuer Index [26]. If liver biopsy was unavailable, liver stiffness measurement by transient elastometry using a cut-off value of 14·6 kPa was acceptable for a diagnosis of cirrhosis [27].

### Determination of HCV RNA

Plasma HCV RNA was determined by a quantitative polymerase chain reaction assay according to the available technique at each center (Cobas AmpliPrep/Cobas TaqMan HCV test v2.0, Roche Diagnostic Corporation, Pleasanton, CA, USA or Roche Diagnostics International AG, Rotkreuz, Switzerland, detection limit: 10 IU/mL; Abbott M2000 Real Time System, Abbott Diagnostic, Chicago, IL, USA; detection limit 12 IU/mL).

### Statistical analysis

Descriptive statistics were conducted for the study population. Continuous variables were expressed as median [interquartile range (Q1-Q3)] and categorical variables as number [percentage; 95% confidence interval (CI)]. The primary efficacy and safety endpoints were SVR12 and the percentage of patients who discontinued therapy due to AEs, respectively. The rate of SVR12 was evaluated in i) an intention-to-treat approach, where all patients were considered and missing values were treated as failures and ii) an on-treatment approach, where those patients who discontinued therapy due to AEs, those who voluntarily dropped out and those who were lost to follow-up were not considered. The association of categorical variables with SVR12 or the proportion of patients who discontinued therapy due to AEs were analyzed using the χ2-test or the Fisher’s test, when applicable. Those factors that showed an association in the univariate analysis with a p<0.2, as well as those with a biologically plausible influence, were entered into a multivariate logistic regression model in order to identify independent predictors for SVR12. The adjusted odds ratio (AOR) and the respective 95% CI were calculated. The positive predictive value (PPV) and the negative predictive value (NPV) of SVR12 in patients achieving undetectable HCV RNA at week four on PI-based therapy were calculated. The latter analysis was restricted to the patients treated according the package inserts of BOC and TVR. Data analysis was conducted using the SPSS statistical software package release 21.0 (IBM Corporation, Somers, NY, USA), STATA 9.0 (StataCorp LP, College Station, TX, USA) and Fisterra.com (Elsevier 2012; http://www.fisterra.com/mbe/investiga/pruebas_diagnosticas/pruebas_diagnosticas.asp).

### Ethical issues

Both study design and conduct conformed to the Helsinki declaration and was approved by the local Ethics Committees of the Valme University Hospital (Comité de Ética de la Investigación Sevilla Sur, Seville, Spain; Ref: 00C3u00A9). All patients gave their written informed consent to participate in the study.

## Results

### Study population

A total of 584 individuals coinfected with HCV genotype 1 and HIV have started BOC or TVR-based therapy at the participating institutions. Of these, 159 patients had reached week 60 of follow-up at the time of analysis (April 2014) and, thus, fullfilled the inclusion criteria for this study: 46 patients received triple therapy based on BOC and 113 individuals were treated with TVR and Peg-INF/RBV. TVR was administered as a bid regimen in ten (8.8%) of these 113 patients. The median (Q1-Q3) age was 48 (42.8–51) years, 127 (79.9%) were male and 45 (34.4%) were treatment-naïve. Assessment of liver fibrosis prior to therapy was available in 133 subjects. Of these, 63 (47.4%) were cirrhotic. The main baseline characteristics of the two groups are shown in Table 1. Five (10.9%) patients received BOC without a lead-in phase and three (2.7%) of those treated with TVR had a four-week lead-in phase prior to TVR initiation. ART was administered in all but one patient (99.4%) and, in the majority of cases, consisted of a combination of tenofovir plus emtricitabine [119 individuals (74.8%)] or abacavir plus lamivudine [20 individuals (12.6%)], along with raltegravir [81 patients (50.9%)] or ritonavir-boosted atazanavir [44 individuals, 27.7%)]. Data on HCV RNA at TW8 was available in 84 patients.

**Table 1:** Baseline characteristics of the patients treated with BOC or TVR.

Parameter	BOC (n = 46)	TVR (n = 113)
Age (years)*	45.4 (41.6–50.9)	48.3 (43–51.4)
Male gender, no. (%)	35 (76.1)	92 (81.4)
IL28B rs12979860 CT/TT, no. (%)^±^	25 (59.5)	70 (70)
HCV subtype 1a, no. (%)^#^	27 (66)	62 (66.7)
Plasma HCV RNA >8*10^5^ IU/mL, no. (%)	37 (80·4)	77 (68.1)
Cirrhosis, no. (%)^¶^	21 (51.2)	42 (45.7)
Liver stiffness	14.8 (6.8–21)	12,3 (7.9–19.5)
Platelets (cells/μL)	178 (119–228)	168 (107–220)
Albumin (g/dL)	4 (3.9–4.3)	4 (3.9–4.2)
Low-density lipoprotein cholesterol (mg/dL)*	103 (77.7–120)	71 (46–102)
Alanine aminotransferase, IU/mL*	62 (39–96)	63 (41–97)
Previous response to anti-HCV therapy^§^		
Naive, no. (%)	19 (41.3)	26 (23)
Null responders, no. (%)	6 (13)	36 (31.9)
Partial responders, no. (%)	0	11 (9.7)
Relapsers, no. (%)	14 (30.4)	19 (16.8)
Other, no. (%)	7 (15.2)	21 (18.6)
CD4 cell count (cells/μL)*	536 (370–670)	630 (414–802)
Undetectable HIV RNA, no. (%)	37 (80.4)	96 (85)

^*^Median (interquartile range);

^±^available in 100 patients receiving TVR and 42 subjects receiving BOC;

^#^available in 93 patients receiving TVR and 41 subjects receiving BOC;

^¶^available in 92 patients receiving TVR and 41 subjects receiving BOC;

^§^as referred to dual therapy with peg-IFN plus RBV**.**

### Response to therapy

**Fig 1:**
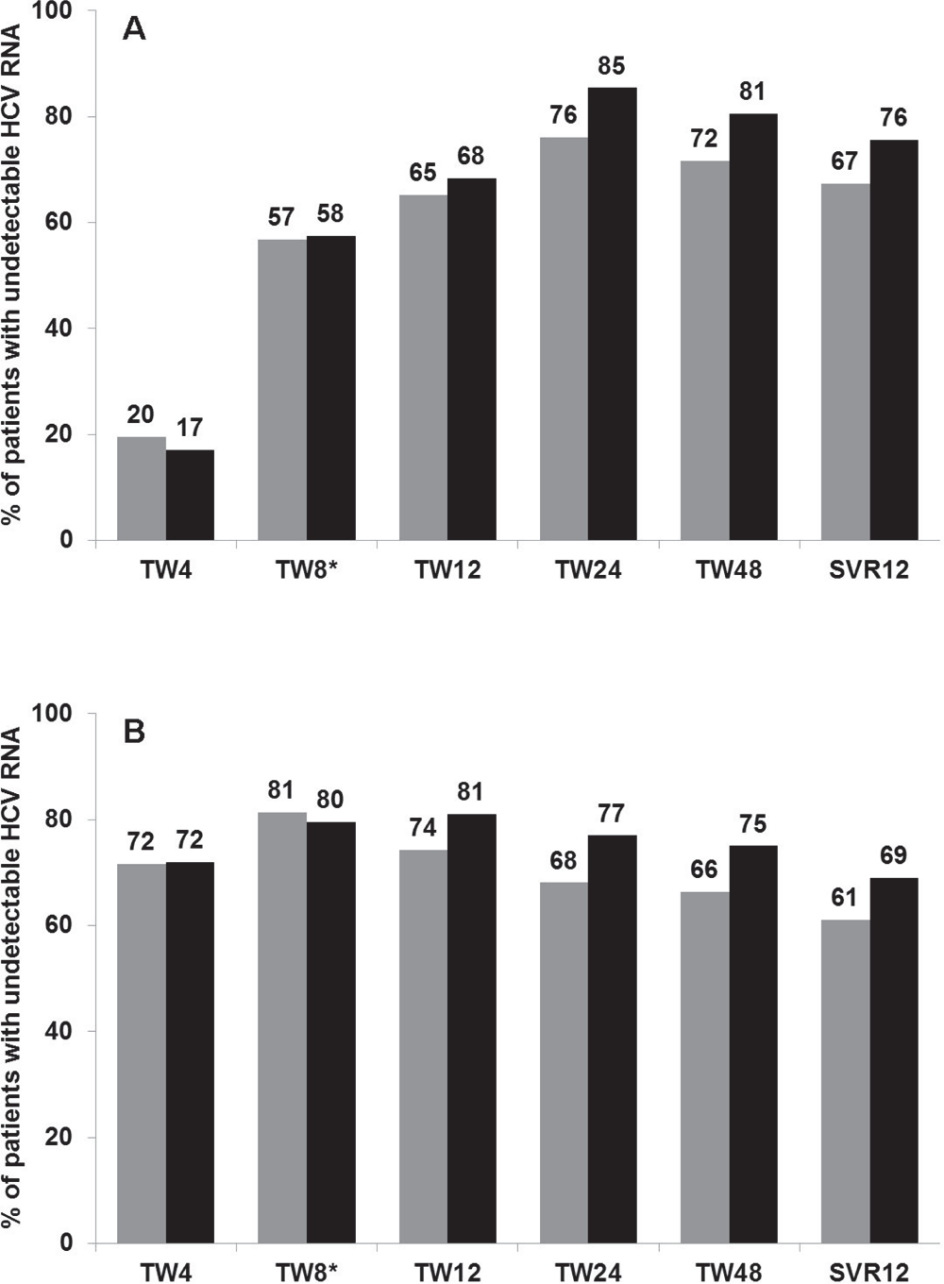
Proportion of patients who presented undetectable HCV RNA at the different treatment weeks (TW). (A) Proportion of patients with undetectable HCV RNA during therapy including peg-IFN plus RBV in combination with BOC. (B) Proportion of patients with undetectable HCV RNA during therapy including peg-IFN plus RBV in combination with TVR. Grey bars: Intention-to-treat analysis; black bars: on-treatment analysis. *TW8 data was available in 44 patients receiving BOC and 43 patients receiving TVR.

The median (Q1-Q3) HCV RNA decline was 1.63 (0.68–2.93) log10 IU/mL after lead-in and 5 (11.4%) subjects had an undetectable HCV-RNA at this time point. A total of 51/159 patients discontinued therapy before reaching the scheduled EOT. Among those who received BOC or TVR, respectively, the numbers were: 5 (10.7%) and 17 (12.8%) met futility rules; 4 (8.7%) and 9 (8%) discontinued due to adverse events; 3 (6.5%) and 8 (7.1%) experienced VB; 1 (2.2%) and 4 (3.5%) voluntary dropped out and 2 (4.3%) and 6 (5.3%) relapsed. A total of 100 patients achieved SVR12, accounting for 62.9% (95% CI: 54.9%-70.4%) treatment success in an intention-to-treat approach (missing = failure): 31/46 (67.4%; 95% CI: 52%-80.5%) of the subjects treated with BOC and 69/113 (61.1%; 95% CI: 51.4%-70.1%) of those treated with TVR. The numbers of patients with an undetectable HCV RNA were 60 (56·6%) at TW4, 60 (69%) at TW8, 114 (71·7%) at TW12 and 112 (70.4%) at TW24, respectively, and 108 (67.9%) patients had an undetectable HCV RNA at the scheduled EOT. The proportions of patients with undetectable HCV RNA at the different treatment points according to the treatment regimen are depicted in Fig 1.

### Safety analysis

**Fig 2:**
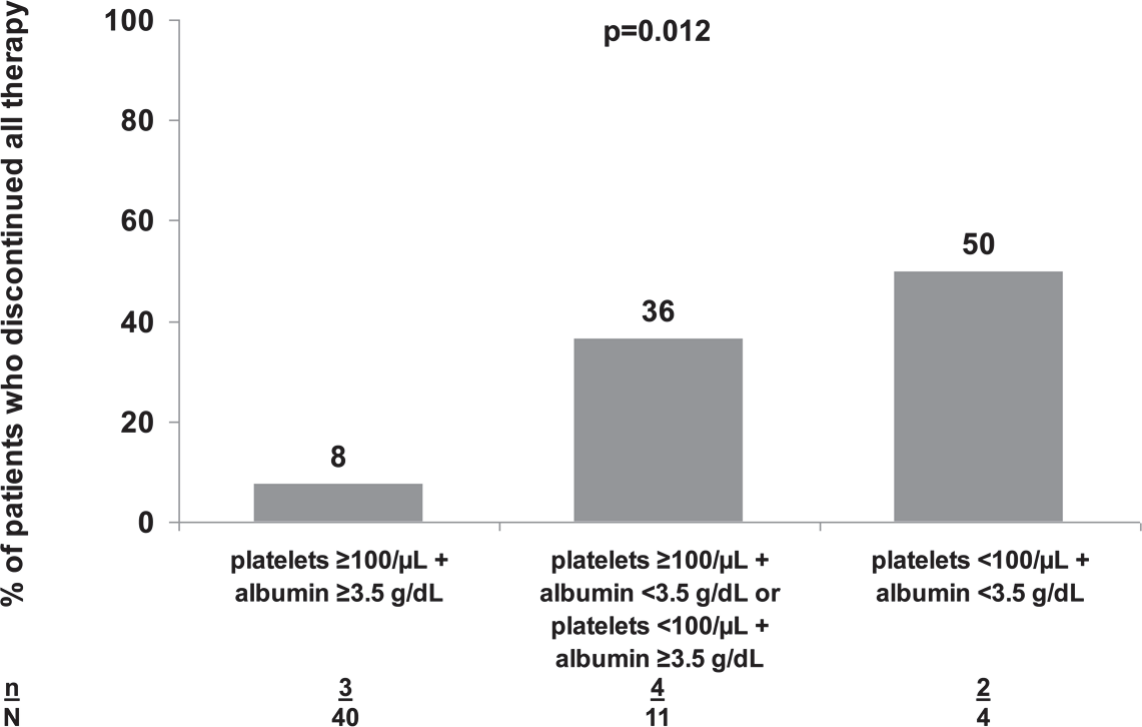
Rate of discontinuations due to adverse events. Proportion of patients who permanently discontinued all therapy due to adverse events after stratifying for baseline platelet count and albumin concentration. Data was available in 46 patients.

Treatment was discontinued due to AEs in 13/159 (8.2%; 95% CI: 4.4%-13.6%) subjects. Any AE were reported in 38/46 (82.6%) of the patients treated with BOC and 92/113 (81·4%) of those treated with TVR. Grade 3 or 4 anemia was reported in 9 (7%) patients, grade 3 or 4 thrombocytopenia was observed in 22 (17.2%) patients and 21 (16.4%) individuals presented grade 3 or 4 neutropenia. Erythropoietin was administered in 36 (22.6%) individuals and nine (5.7%) received blood transfusion. General dose reductions of Peg-IFN and RBV were reported in 16 (17.4%) and 40 (43.5%) patients of the 92 subjects respectively in whom these data were available. Table 2 summarizes detailed hematological abnormalities observed in the subgroups receiving BOC- or TVR-based therapy. Four (2.5%) individuals developed hepatic decompensation: two (4.3%) individuals treated with BOC and two (1.8%) treated with TVR. One of these patients died due to hepatic failure at TW24 on triple therapy with BOC. Two treatment discontinuations due to AEs to BOC-based therapy were due to severe hematological abnormalities and one due to development of ascites. The AEs leading to early treatment discontinuation in those who received TVR-based therapy were: hematological abnormalities (three patients), depression (three patients), hepatic encephalopathy (one patient), gastrointestinal bleeding and severe skin rash (one patient). Discontinuations due to AEs was observed in 6/73 (8.2%) of those with cirrhosis at baseline versus 7/60 (11.7%) of those without (p = 0.505). Likewise, discontinuations due to AEs was observed in 4 (3.7%) out of 109 patients with a platelet count ≥100 cells/μL versus seven (25%) of the 28 subjects with a platelet count of <100 cells/μL (p<0.001) and in 7/50 (14%) versus 2/6 (33%) of those with baseline albumin values above or below 3.5 g/L (p = 0.223), respectively. Fig 2 depicts the additive effect of baseline platelet count and albumin concentration on discontinuations due to AEs.

**Table 2:** Hematological abnormalities observed among patients receiving BOC- or TVR-based triple therapy and actions taken.

Event, n (%)	BOC (n = 46)	TVR (n = 113)
Anemia (hemoglobin mg/dL)*		
9.5–10.9	16 (37.2)	28 (32.9)
8–9.4	3 (7)	11 (12.9)
<8	5 (11.6)	4 (4.7)
Thrombocytopenia (platelets/μL)*		
70000–99000	8 (18.6)	18 (21.2)
50000–69000	10 (23.3)	12 (14.1)
<50000	7 (16.3)	15 (17.6)
Neutropenia (neutrophils/μL)*		
1000–1499	11 (25.6)	26 (30.6)
750–999	7 (16.3)	3 (3.5)
<750	7 (16.3)	14 (16.5)
Use of erythropoietin	22 (47.8)	14 (12.4)
Blood transfusion	5 (10.9)	4 (3.5)
Dose reduction of ribavirin^±^	28 (66.7)	63 (67.7)

^*^detailed hematological data was available for 85 patients receiving TVR and 43 subjects receiving BOC;

^±^information available in 93 patients receiving TVR and 42 subjects receiving BOC.

### Predictors of response

The analysis of predictive factors for treatment success was carried out with regard to SVR12 in an on-treatment approach. A total of 24/36 (66.7%) patients who received ritonavir-boosted atazanavir and 53/74 (71.6%) individuals who received raltegravir achieved SVR12 (p = 0.821). The rates of SVR12 (n/N patients) according to HCV subtype 1a and 1b were 71.8% (56/78) versus 70% (28/40) (p = 0.839). Seventy-four (71.8%) out of 103 subjects with a platelet count of ≥100 cells/μL at baseline versus 11 (57.9%) of 19 patients with <100 cells/μL at baseline achieved SVR12 (p = 0.224). All four patients who had plasma albumin levels below 3.5 g/L versus 26/42 (61.9%) of those with baseline albumin values higher than 3.5 g/L achieved SVR12 (p = 0.126). The relationship between other possible predictors and SVR12 is shown in Table 3. In the multivariate analysis, previous response to dual therapy and lower baseline HCV RNA levels were independently associated with SVR12 (Table 3). Among those patients who achieved undetectable HCV RNA four weeks after HCV PI initiation, 97 (86.6%) achieved SVR12 versus 16 (36.4%) of those who had detectable viral load at week four (p<0.001). The PPV to predict SVR12 in patients achieving undetectable HCV RNA at week four was 85·5% (95% CI: 75.2%-92.2%) while the NPV was 49.2% (95% CI: 36.1%-62.4).

**Table 3:** Univariate and multivariate analysis to identify factors associated with sustained virologic response 12 weeks after scheduled end of therapy (SVR12).

Parameter	SVR12,	P	AOR	p
	n (%)	univariate	(95% CI)	multivariate
Age				
≤48 years	50 (68.5)	0.511	0.983 (0.906–1.066)	0.672
>48 years	50 (73.5)		1	
Gender				
Male	82 (70.7)	0.896	0.563 (0.417–2.157)	0.402
Female	18 (72)		1	
Baseline cirrhosis^±^				
No	47 (73.4)	0.279	1	
Yes	34 (64.2)		0.936 (0.306–2.865)	0.908
Baseline HCV RNA				
<800 kIU/mL	33 (84.6)	0.027	11.959 (2.145–66.69)	0.005
≥800 kIU/mL	67 (65.7)		1	
*IL28B* rs12979860				
CC	33 (84.6)	0.025	1.741 (0.477–6.361)	0.401
CT/TT	58 (65.2)		1	
Previous response^¶^				0.022
Naïve	26 (74.4)	0.01	1	
Relapse	25 (92.6)		6.799 (0.698–66.248)	0.099
Partial response	6 (66.7)		3.165 (0.298–33.576)	0.339
Null response	22 (55)		0.316 (0.094–1.062)	0.062

AOR: adjusted odds ratio; CI: confidence interval; *IL28B*: interleukin 28B.

^±^available in 100 patients receiving TVR and 42 subject receiving BOC;

^¶^as referred to dual therapy with peg-IFN plus RBV.

## Discussion

The results of this study show that the efficacy of triple therapy including BOC or TVR in combination with peg-IFN/RBV plus RBV in patients coinfected with HIV and HCV genotype 1 under real life conditions is comparable to that observed in clinical trials, both in HCV-monoinfected [4–7] and in HIV/HCV-coinfected [8,9] subjects. The described response rates are very well in line with the new guidelines of the European Association for the Study of the Liver (EASL) [13] which no longer separate between HIV/HCV and HCV monoinfected patients with regard to indication and choice of therapy, other than drug-drug interactions, due to similar cure rates in the two populations. In addition, these therapies are tolerable for most patients; indeed, the number of discontinuations due to AEs is slightly lower than that previously reported for dual therapy with Peg-IFN plus RBV [28,29].

A number of studies have evaluated the efficacy of first-generation PIs [16–19], however, data are derived from different populations and are thus not comparable since they differ in the proportion of patients with advanced liver damage, ethiology and social background that may impact on the adherence. The CUPIC study (compassionate use of BOC and TVR in France) reported SVR12 rates of 42.9% and 51.8%, respectively, in a treatment-experienced, cirrhotic population [16]. Furthermore preliminary results obtained from the expanded access study HEP3002 suggest that the efficacy of TVR in patients with advanced fibrosis treated in the clinical practice is comparable to that observed in clinical trials [17]. Importantly, and in contrast to the present study, these data were obtained from HIV (-) patients. Data on the use of HCV PIs in HIV/HCV-coinfected patients in clinical practice are scarce and mainly available for TVR-based triple therapy [30,31]. The present study includes a large number of HIV/HCV-coinfected patients derived from various centers throughout Europe. Most were treated with TVR, of whom 61% achieved SVR12 in an intention-to-treat analysis. This is a somewhat lower response when compared to phase II clinical trials conducted in treatment-naïve HIV/HCV-coinfected patients, with a low frequency of cirrhosis [9,15]. This is not surprising, since this study includes a higher proportion of difficult-to-treat subjects; specifically individuals bearing advanced fibrosis or cirrhosis and previous non-responders to dual therapy. These findings are, however, consistent with interim and SVR12 data reported from studies conducted in HIV/HCV-coinfected patients [30–32]. With regards to those who received BOC-based triple therapy in the present study, a SVR12 rate of 67% was observed. It is likely that the high number of previously naïve patients and relapsers included herein accounts for this comparably high response rate.

The rates of discontinuations due to AEs were 8% in the present study and thus somewhat lower than those reported in clinical trials [8,9,14,15] and even lower than that observed with dual therapy with historical Peg-IFN/RBV in some of the centers participating in the study [28,29]. This probably represents increased tolerability of medication associated side-effects in patients accessing new therapy. In this context, rates of discontinuations due to AEs have improved over time when compared within the same cohorts [28,29]. AEs, predominantly flu-like symptoms, were reported in the majority of the patients. Despite the high proportion of cirrhotic patients, only four individuals developed hepatic decompensations and the rate of discontinuations due to AEs was not influenced by baseline cirrhosis. On the other hand, and in accordance with previously reported data from the CUPIC study [16], baseline platelet count and albumin levels have an impact on the likelihood of discontinuations due to AEs. These observations suggest that although AEs are frequently observed, they are manageable, and that discontinuations rates due to AEs are not higher to what was observed with dual therapy. On the other hand our population was also highly motived as they were waiting for the first generation PI since a long time and hence they were struggling hard not to stop therapy before week 48.

The present analysis shows that treatment response four weeks after initiation of the HCV PI has a high PPV for SVR12 in HIV/HCV-coinfected patients, in real life conditions, including previous non-responders and cirrhotic patients. The role of response in the first weeks of BOC- or TVR- based therapy as predictor of treatment response had been previously described in HCV-monoinfected patients with cirrhosis [33]. In fact, response-guided therapy in HIV/HCV-coinfected patients can be applied in TVR-based therapy in previous relapsers or treatment-naïve patients without cirrhosis [24,25]. Likewise, the role of response-guided therapy in BOC-based regimens is currently being evaluated in HIV/HCV co-infected patients [34,35]. Our results suggests that response-guided shortening of therapy duration should be evaluated not only in naïve or relapsing patients, but might be also appropriate in harder-to-cure subjects.

Previous relapsers showed highest SVR12 rates when compared to treatment-naïve subjects and previous null or partial responders, while the latter show the poorest response to therapy. This is in accordance with data obtained from clinical trials conducted in HCV-monoinfected and HIV/HCV-coinfected patients [5,7], as well as the UNITE trial [10]. On the other hand, a diagnosis of cirrhosis did not significantly influence the achievement of SVR12. The reason for this finding could be that in most patients cirrhosis diagnosis was based on liver stiffness measurement or biopsy, both allowing an earlier diagnosis than clinical or biological data [36]. Late-stage cirrhosis usually affects treatment outcome. However, in this study subjects were treated with ITF-based combinations. Consequently, the vast majority of the cirrhotic patients presented compensated cirrhosis and, in this setting, the impact on SVR could be less obvious. Nevertheless, there was a difference of 9 percent-points between subjects who presented F0-F3 versus those with F4 at baseline. It is therefore likely that a higher sample size would have led to a statistically significant impact of cirrhosis on treatment outcome. However, for patients with biological abnormalities consistent with advanced cirrhosis and portal hypertension, such as hypoalbuminemia or thrombocytopenia, the rate of adverse effects was higher.

The present work has a certain limitations. The clinical impact of this work might diminish since next generation DAAs show improved efficacy over TVR or BOC, show better tolerability, a lower potential of DDIs and are easier to administer [37,38]. However, these regimens may not be widely used in the next years, because of financial constraints even in developed countries. Therefore, triple therapy including BOC or TVR may well remain of importance in the near future. Additionally, this study represents an important historic reference to evaluate the evolution of SVR rates to different regimens over time under real-life condition in the coinfected population.

In conclusion, the efficacy and safety of triple therapy including BOC or TVR in combination with Peg-IFN plus RBV in routine clinical practice in a HIV/HCV-coinfected population was acceptable and comparable to what is observed in clinical trials. Drug-related toxicity can be managed and AEs do not lead to discontinuations in the majority of the patients.
